# Clinical Thermometry

**Published:** 1875-06-15

**Authors:** B. Kraus


### Translations.

### CLINICAL THERMOMETRY.

FROM THE "COMPENDIUM DER NEUEREN MEDICINISCHEN WISSENSCHAFTEN," BY DR. B. KRAUS.

*Condensed and translated by Dr. H. Gradle.*

IN small-pox, either variola vera or a complication must be diagnosed, if the temperature remains febrile after the appearance of the eruption. In idiopathic croupous and lobar pneumonia the observation of a normal temperature at a certain time is not absolute evidence of the completion of the morbid process. A *very* high rise of the mercury denotes a great intensity or extension of the inflammation. Facial erysipelas threatens to extend further, and complications are to be feared as long as the temperature remains febrile in that affection. In bronchitis and influenza, a *highly* febrile condition shows involvement of the capillary bronchial tubes, or commencement of a pneumonitis; it occurs also in caseous degeneration of the exudation. Pertussis is always complicated when the temperature is abnormal. In acute rheumatism the height of the mercury is an index of the danger and probability of complications.

In meningeal and cerebral disorders the thermometer is of less diagnostic value. Wunderlich thinks that involvement of the convexity of the hemispheres is accompanied by a higher temperature than basilar meningitis, in which latter the normal heat may not be exceeded. As is well known, the pulse indicates by its acceleration an irritated condition of the brain; by its slackening a compression of the same, but the exact seat of the disorder is not thus revealed. The same seems to be the case with the thermometric evidence.

Inflammations of serous membranes increase in danger according to the height of the temperature, yet a moderate fever does not always warrant a favorable prognosis.

Repeated observation of a high temperature, with the co-existence of gastro-intestinal catarrh, indicates the occurrence of severe disease, as typhoid, etc.

Wunderlich doubts the existence of intermittent fever, if at the beginning of the hot stage the mercury reaches the vicinity of 41° C. (106° F.) or exceeds 41.8° C. (107.25° F.); but if the apyrexia is imperfect, the disease is again questionable. If, after the attacks have ceased, the temperature undergoes still abnormal exacerbations, the disease is not cured. In severe cases of this disease, especially in feeble and irritable individuals, the decline of the temperature towards the close may fall much below the normal limits, and be accompanied—or not—by other symptoms of collapse, without, however, indicating danger, if this collapse corresponds to the period of defervescence.

### TEMPERATURE DURING CONVALESCENCE.

During convalescence the body may possess its normal degree of heat, but after the occurrence of severe, especially wasting, diseases, the temperature is usually below the normal degree; sometimes it is higher. Accordingly convalescence has been designated normal or irregular. A high temperature is generally owing to imperfect convalescence, while collapse, if considerable, is also not without danger, and points often to internal hæmorrhage, intestinal perforation, etc. Yet a temperature but slightly less than normal is not a dangerous sign, merely indicating that nutrition does not yet correspond to the demands of the system.

During convalescence the temperature possesses still an abnormal instability, hence a sudden febrile rise is not very rare. This may be due to errors in diet, too early partaking solid food, or spirits, or an excessive meal, or too great exertion from early rising, etc. Often a rise occurs immediately on leaving the bed. Constipation and other unimportant circumstances, are also of marked influence on the temperature. Yet the cause of the rise of temperature may also be a serious disorder, revealing itself at the time merely by that symptom, hence an unexpected result of a single thermometric observation should always receive due consideration.

### TEMPERATURE AFTER DEATH.

After death the temperature shows some interesting alterations; in most cases it falls the more rapidly the lower the animal heat at the moment of death. Alvarenga details the thermometric course in the case of a man aged 58 years, who entered the clinic with cerebral apoplexy, and died of the same after the lapse of fourteen hours. The thermometer showed, one hour before death, 36.5° C., and one quarter of an hour after death, 35.6° C. Hereupon, retaining the instrument in the axilla, the following numbers were read off, record being taken every *fifteen minutes:* 35.6, 35.4, 35.0, 34.6, 34.0, 33.4, 32.8, 32.2, 31.6, 31.0, 30.6, 30.0, 29.6, 29.0, 28.4, 27.0, 25.6, 23.8, 20.6, 18.0, 15.8, 15, stationary.

Occasionally the temperature rises *post-mortem*, surpassing even the maximum during life. Jaccoud (*Traite de la path. interne*, T. 1) mentions that when a slight rise occurs, of but a few tenths degree, it takes place about an hour after death, whereupon, after the temperature has remained stationary for a short time, it begins to fall, at first slowly and thereupon more rapidly; if, however, the *post-mortem* rise has been considerable, the decline is much slower to occur, as in tetanus, cholera, and other febrile disorders, attended with an increase up to the last moment. The cause of the rise of temperature has been sought in (1) the suspension of respiration and cutaneous secretion, sources of loss of heat; (2) the uninterrupted continuance of the chemical processes, the means of production of heat, and (3) the *post-mortem* changes in muscular tissue, considered also a source of heat.

### THE COURSE OF PATHOLOGICAL TEMPERATURE.

The normal diurnal oscillations of temperature become vastly more considerable in disease, so as to amount usually to 1–1½°C., and even 5–6° difference between the daily minimal and maximal temperature. The extent of the diurnal oscillations depends on the nature, intensity, and stage of the disease, the complications and accidents, the favorable or fatal tendency of the affection, as well as on individual circumstances and therapeutic measures. The particular thermometric course of a disease is often sufficiently distinct to serve as means of diagnosis of that disorder.

The temperature either remains abnormally elevated *constantly*, until the acme is passed, giving the type of a continuous fever, or it returns at periods to its normal height, to describe thereafter a similar curve—intermittent fevers. In the latter class, each period of pyrexia can be considered as a special course of fever, a repetition of which is to be expected until the disease is cured.

The thermometric course of a disease consists of different stages characterized by a special thermometric curve of their own, sometimes abruptly, more often gradually, passing into each other. The *pyrogenetic* or initial stage, differs according to whether the fever is dependent on a local disease or not, and whether it precedes or follows the latter. In the former case the fever begins abruptly and reaches a high grade before the local disturbances are manifest; in such cases the initial stage is said to be completed, when the lowest mean temperature of the day characteristic of that disease is attained, or when the local symptoms begin to appear. In fevers without localization the first stage is not distinct, and the limits between it and the second are arbitrary.

The variations of disease, as regards their initial stage, are classified by Wunderlich under three heads: *a.* Diseases with short pyrogenetic stage, the temperature describing an almost straight, oblique line. *b.* Affections with initial stage of several days' duration, the temperature usually rising in the evening and sinking again in the morning, at which time it may be even normal. Occasionally this stage is interrupted by continuous apyrexia. *c.* Diseases with still more gradual development, generally irregular and not distinctly typical in their course.

Differences in temperature during the height of disease, the second stage, may occur as regards the absolute maximum, dependent on the nature and intensity of the affection, but too much weight ought not to be placed on this, unless of such a height as to threaten life by itself, since a temporary high temperature may result from various extraneous influences. Of more importance are the differences in the diurnal average during the acme, the mean of which for the entire period is still more characteristic. But the most valuable basis for decision in any case is the entire thermometric course during the acme. This may be ascending to a point at which either death occurs, or a rapid decline commences, or it may remain stationary at a certain height, undergoing merely slight diurnal oscillations of about $\frac{1}{2}°$, or finally be irregular. This second stage may terminate abruptly, or gradually pass into another period. It is often followed, especially in favorable cases passing through a severe course, by a period of unsteadiness of temperature.

The thermometric course during the process of repair is divided by Wunderlich into the following periods: *a.* The stage of decided but insufficient decline; *b.* defervescence;

*c.* the period following defervescence, and *d,* convalescence. The stage of decline is not present in all cases; occasionally it commences with a short precursory *rise* of temperature. The period of defervescence is the most distinct stage in different diseases, and deviations from its typical course are good evidence of anomalies or imperfect repair. The course of defervescence constitutes either *crisis,* in which the temperature falls 2–5° C., or more, within thirty-six hours at the latest, reaching the normal height, or even sinking beyond it; or it is termed *lysis* when the decline is much more gradual. In this mode of defervescence the temperature shows a greater decline from evening to the next morning than from the latter period to the consecutive evening, during which time it may remain stationary or even rise slightly. In this way the period may be protracted two to four days, or even one week, as we see in scarlatina, in typhus, occasionally in pneumonitis of long duration; rarely does this occur in typhoid fever, oftener in catarrhal fevers. The course of lysis may also be remittent, considerable morning remissions alternating with high evening exacerbations while on the whole the daily average and maximum steadily decreases.

A consecutive disease begins to show its influence on the temperature after the latter has passed through the usual course to convalescence, provided it is of a febrile nature.

The fatal issue is preceded by thermometric phenomena, which, in some cases might be interpreted as apparent improvement; in others, however, as of decided unfavorable prognostic evidence; and but rarely do we succeed in such cases in avoiding the fatal issue by powerful interference. This stage, preceding the moribund state, differs in its thermometric course from both the ordinary course of the disease and the true agony. According to Wunderlich, the following varieties of this stage may occur:

*a.* The ascending form, in which, with the exception, perhaps, of slight morning remissions, the temperature rises steadily until the occurrence of agony or death itself. Its beginning is more or less distinct, according to the height of the fever existing previously.

*b.* The descending form, much more frequent, in which the decline of temperature might be taken as a favorable sign if it were not for the concomitant symptoms, especially the enormous rise in the frequency of the pulse. This stage is mostly of short duration, from one-half to two days, the temperature sinking only about 1° C., though sometimes reaching the normal height. Not unfrequently the temperature begins to rise again in the following stage of agony.

*c.* In some cases this stage is indicated only by other symptoms, for instance, the pulse, and not at all by any thermometric change, especially when cyanosis is present, though, in the latter case, the descending form is the more common.

*d.* The last variety is characterized by great oscillations of temperature, high exacerbations alternating with very low remissions, as is the case in pyæmic disorder, and desperate affections which have been treated very energetically.

[*To be continued.*]

